# Automatic measurement of short-term variability of repolarization on human intracardiac electrograms predicts VT/VF events

**DOI:** 10.1016/j.hroo.2026.06.005

**Published:** 2026-06-16

**Authors:** Alfonso Aranda Hernández, Mathias Meine, Marc Vos

**Affiliations:** 1 Cardiac Rhythm Management, Medtronic, Mounds View, Minnesota; 2 Department of Cardiology, University Medical Center Utrecht, Utrecht, The Netherlands; 3 Department of Medical Physiology, University Medical Center Utrecht, Utrecht, The Netherlands

**Keywords:** Short-term variability, Repolarization, Ventricular arrhythmia, Electrogram, Implantable cardioverter-defibrillator

## Abstract

**Background:**

Implantable cardioverter-defibrillators (ICDs) have proven highly effective in reducing sudden cardiac death by terminating life-threatening arrhythmias. However, their reactive nature underscores the need for preventive strategies to further improve patient outcomes. Short-term variability (STV) of repolarization has emerged as a promising predictive marker for ventricular arrhythmias. Despite its potential, the feasibility of implementing STV monitoring in real-world ICD settings remains uncertain owing to challenges such as noise, abnormal depolarizations, and device-specific constraints.

**Objective:**

This study aimed to evaluate the feasibility of STV monitoring in clinical practice using anonymized electrogram recordings from Medtronic Cobalt/Crome ICD and cardiac resynchronization therapy defibrillator devices.

**Methods:**

The dataset includes 636 monomorphic ventricular tachycardia episodes, 107 ventricular fibrillation events, and 6000 baseline recordings. Cardiac beats with abnormal repolarization patterns or noise were excluded to ensure reliable STV estimation. STV values were then compared within individuals (baseline vs arrhythmic events) and across groups (patients with and without arrhythmic events).

**Results:**

STV values were significantly elevated before arrhythmic events compared with baseline 5.8 ms (interquartile range, 3.3–7.6) vs 4.3 ms (interquartile range, 2.8–7.6) (*P* < .0001). These findings support STV’s potential as a predictor of ventricular arrhythmias. However, the precise timing of STV elevation relative to arrhythmic onset remains unclear.

**Conclusion:**

This study supports the role of STV as a marker for arrhythmic risk, evidenced by significant elevations in STV preceding arrhythmic events. It also emphasizes the importance of signal preprocessing to mitigate confounding factors and enhance the predictive accuracy of the STV parameter.

**Key Findings**

▪ Short-term variability (STV) derived from intracardiac electrogram recordings increased significantly preceding arrhythmic events compared with baseline recordings.
▪ Both signal noise and premature ventricular contractions substantially increased STV values, thereby affecting STV discriminative performance.
▪ Continuous STV monitoring may provide additional value for arrhythmic risk stratification in patients with implanted cardiac devices.

## Introduction

Over the past century, the management of ventricular arrhythmias and the prevention of sudden cardiac death (SCD) have significantly improved, driven by continuous innovations in diagnostics, risk stratification, and therapeutic interventions. Among these, the introduction of the implantable cardioverter-defibrillator (ICD) represented a landmark achievement by providing effective, lifesaving therapy for individuals at high risk of fatal arrhythmic events.^1^^,^^2^ The widespread adoption of ICDs has led to substantial reductions in SCD-related mortality, particularly in patients with structural heart disease, inherited arrhythmic syndromes, and heart failure.^3^ Despite these advances, SCD remains a public health challenge, accounting for a significant proportion of cardiovascular deaths worldwide.^4^ Although ICDs offer crucial protection, they primarily serve as a reactive therapy rather than a preventive measure against the underlying electrophysiological mechanisms that predispose patients to malignant arrhythmias. Developing new predictive and preventive approaches to complement ICD therapy has the potential to further reduce the burden of SCD and improve patient outcomes.^5^

Among the various arrhythmic risk markers explored in the scientific literature, the assessment of temporal variability of repolarization via beat-to-beat variability has emerged as an important marker of electrical instability. Over the past decades, several markers have been proposed to quantify these phenomena, including T-wave alternans,^6^^,^^7^ periodic repolarization dynamics,^8^ QT interval variability,^9^ QT integral,^10^ and activation–recovery interval variability.^11^ Although these approaches differ in methodology and temporal scale, they share the common objective of capturing dynamic fluctuations in ventricular repolarization that may reflect increased electrical instability and arrhythmic susceptibility.

Short-term variability (STV) in repolarization has emerged as a marker of beat-to-beat repolarization instability. Experimental studies in animal models have demonstrated that STV effectively differentiates individuals who later develop ventricular arrhythmias or experience SCD over time.^12^^,^^13^ This predictive capability has also been observed in clinical populations, including patients with acquired^14^ or congenital^15^ long-QT syndrome, nonischemic heart failure,^16^ and structural heart disease^17^ and primary prophylactic ICD patients.^18^ In these patient groups, an elevated STV value was associated with a higher likelihood of arrhythmias. These findings suggest that STV may serve as a valuable tool for identifying individuals at higher arrhythmic risk, enabling more targeted monitoring and intervention strategies.^19^^,^^20^

One of the main challenges for automatic continuous monitoring of STV in patients during daily activities is the potential interference from muscle noise, electromagnetic interference, and other artifacts in electrocardiogram and electrogram (EGM) recordings. These factors can confound STV values, leading to erroneous interpretations that are unrelated to actual increases in arrhythmic risk. Furthermore, the accuracy of STV is highly sensitive to sampling frequency and amplitude dynamic range. This sensitivity poses a significant limitation for its implementation in cardiac devices, which often operate with constrained sampling rates and limited dynamic ranges. As a result, the feasibility of reliably measuring STV in such devices may be restricted or, in some cases, impractical.

Given these challenges, it is essential to explore whether STV can still provide meaningful insights when measured in cardiac devices and in clinical settings. In an effort to better understand the feasibility of STV monitoring in cardiac devices, we analyzed anonymized data from Medtronic ICD devices and cardiac resynchronization therapy defibrillator (CRT-D) devices. We assessed whether baseline transmissions exhibit lower STV values than subsequent transmissions preceding arrhythmic events. This study provides critical insights into the potential clinical utility of STV monitoring, helping to determine its feasibility for arrhythmic risk assessment in patients with implanted cardiac devices.

## Methods

### Database

The database comprises EGM signals from Medtronic Cobalt/Crome CRT-D and ICD devices, containing recordings associated with both baseline conditions and arrhythmic events, such as monomorphic ventricular tachycardia (MVT), polymorphic ventricular tachycardia (PVT), ventricular fibrillation (VF), supraventricular tachycardia (SVT), atrial tachycardia (AT), and atrial fibrillation (AF).

**Figure 1:**
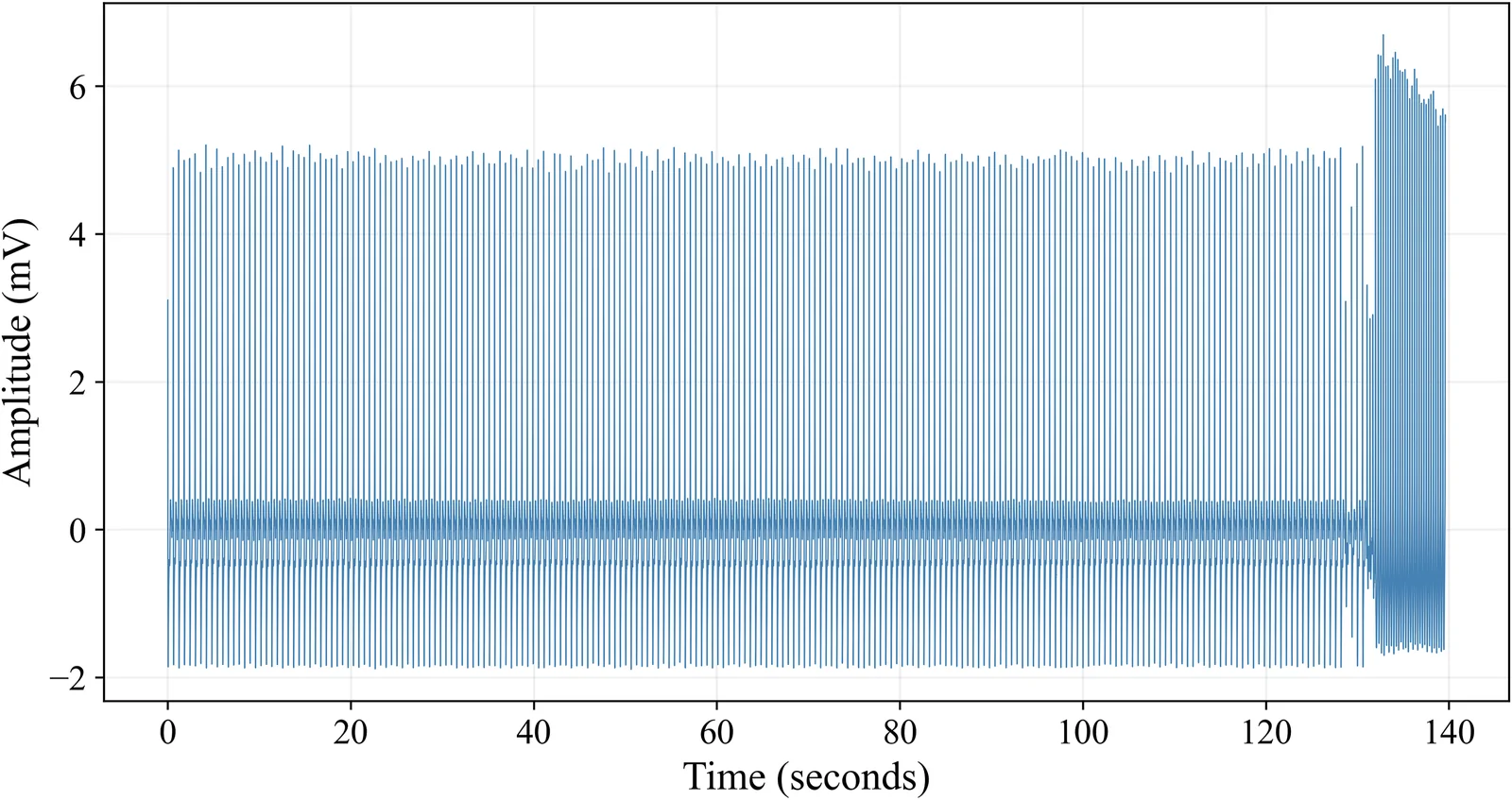
Example of an electrogram recording with a monomorphic ventricular tachycardia event in the database.

In this study, only MVT, PVT, and VF device-treated episodes were analyzed, whereas other treated arrhythmic events (eg, SVT, AT, AF) were excluded. Each transmission has a duration of ∼140 seconds, with EGM signals sampled at 256 Hz and recorded with 16-bit resolution. A typical MVT recording within the database is presented in Figure 1.

In the database, there are 636 MVT episodes, 107 PVT/VF events, and 6000 baseline recordings without associated MVT/PVT/VF occurrences. It is worth noting that the absence of arrhythmic events in baseline recordings does not imply that these patients will remain event-free in the future. We categorized patients into 2 distinct groups:

• Group 1: patients without recorded arrhythmic events.
• Group 2: patients with recorded arrhythmic events.

For both groups, recordings were excluded from the analysis if they exhibited more than 10 abnormal beats (ABs), presented excessive noise, or showed AT or AF. ABs were defined as premature ventricular contractions, premature atrial contractions, and any other beats with repolarization morphologies differing from normal beats, which could introduce confounding in STV measurements.

**Figure 2:**
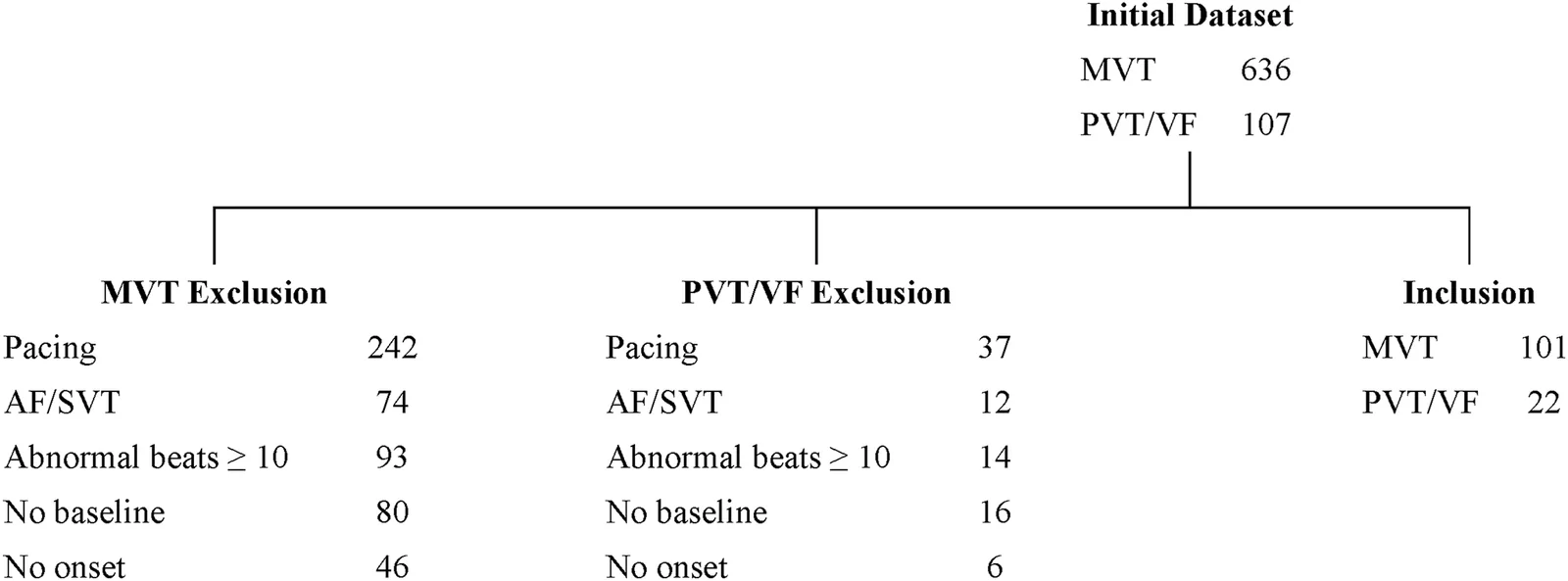
Flowchart depicting the exclusion criteria applied to the dataset. AF = atrial fibrillation; MVT = monomorphic ventricular tachycardia; PVT = polymorphic ventricular tachycardia; SVT = supraventricular tachycardia; VF = ventricular fibrillation.

Furthermore, recordings containing pacing were discarded in our analysis. The reasons were as follows: (1) the existing body of evidence on STV is based on intrinsic rhythm conditions, (2) mixing pacing and intrinsic conditions could introduce variability in STV measurements (owing to different beat morphologies) that might not be associated with arrhythmic risk, and (3) pacing induces physiological changes in the EGM making STV changes under pacing conditions not comparable with those obtained during intrinsic rhythm.^21^ The applied exclusion criteria and the corresponding reduction in available recordings are presented in Figure 2. After exclusions, 101 MVT and 22 PVT/VF episodes were included in the analysis.

### Annotation process

The annotation process involved 3 key steps:

1. Episode classification: episodes were manually reviewed through visual inspection. Each episode was classified into 1 of the following categories:
  a. Normal: intrinsic rhythm episodes associated with normal transmissions and not containing arrhythmic events.
  b. MVT: episodes consistent with MVT.
  c. PVT/VF: recordings showing PVT or VF.
  d. Other: this category included AT/AF, SVT, nonsustained ventricular tachycardia, bigeminy, and any other rhythms not classified as normal, MVT, or PVT/VF.
2. AB detection: ABs were manually reviewed and annotated through visual inspection.
3. Noise detection: noise detection was performed automatically using the algorithm described in the section "Noise-detection algorithm". This algorithm identified and flagged sections of the recordings affected by excessive noise, ensuring that only high-quality data were included in subsequent analyses.

### Noise-detection algorithm

To automatically detect noise in the EGM and discard values when excessive noise is present, we developed a relatively simple method. The method consists of filtering the signal using a Savitzky–Golay filter, computing the residual signal, and performing a convolution operation.

The first step involves filtering the original signal using a Savitzky–Golay filter, selected for its effectiveness in attenuating high-frequency noise while preserving signal shape. Based on empirical testing, we used a polynomial order of 5 and a window size of 21 samples. The aim of using the filter is to remove noise in the signal while preserving important features such as peak height and width. Then, the original and filtered signals are subtracted and rectified to get an estimation of noise as:

(1) $$Noise=\left|EGM-{EGM}_{filtered}\right|$$

**Figure 3:**
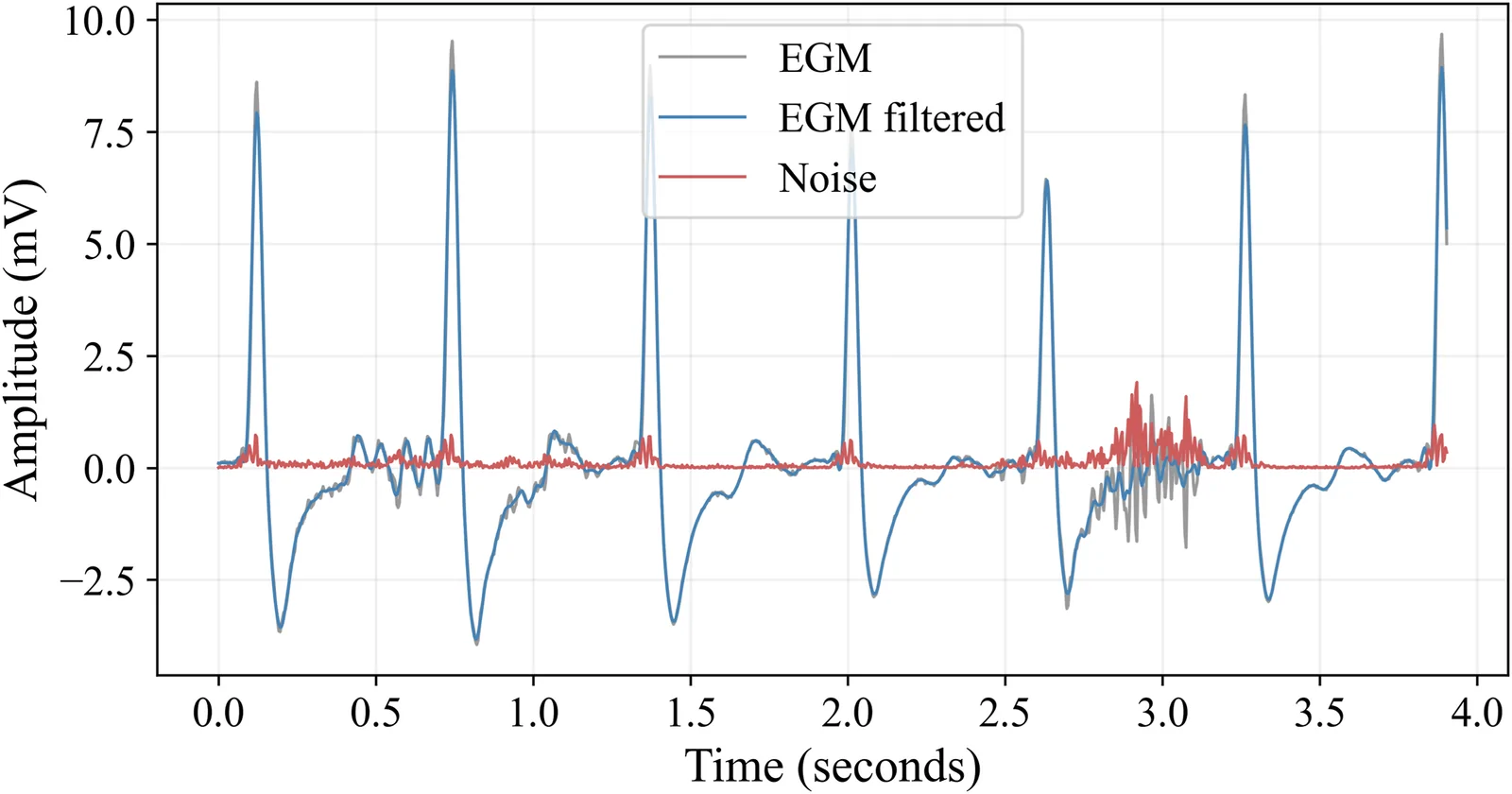
Noise signal estimation using Savitzky–Golay filtering. EGM = electrogram.

An example where “EGM” reflects the original EGM signal (gray color), “EGM filtered” shows the filtered signal (blue line), and “Noise” depicts the estimated noise using equation 1 (red line) is presented in Figure 3.

Once we have an estimation of the noise in the EGM signal, the amount of noise in each cardiac cycle is estimated as follows:

(2) $${Noise}_{beat}=\frac{\sum_{i=60}^{p}{Noise}_{i}}{p-60}$$

where *Noise*_*i*_ depicts the noise estimation for a given sample point in a cardiac cycle and *p* is the number of samples of the cardiac cycle. The summation starts at 60 (∼234 ms at 256 Hz) and extends to *p* to exclude the first *60* samples, thereby preventing R-wave filtering effects from being misclassified as noise. The division by *(p – 60)* normalizes the noise estimation, providing a representative measure of noise per sample within the cardiac cycle. Next, a convolution operation using a 31-beat window is applied to obtain the convolved noise signal (*Noise*_*conv*_). This step is essential because an increase in noise during a single beat can influence the subsequent 32 STV value calculations, potentially confounding the analysis.

**Figure 4:**
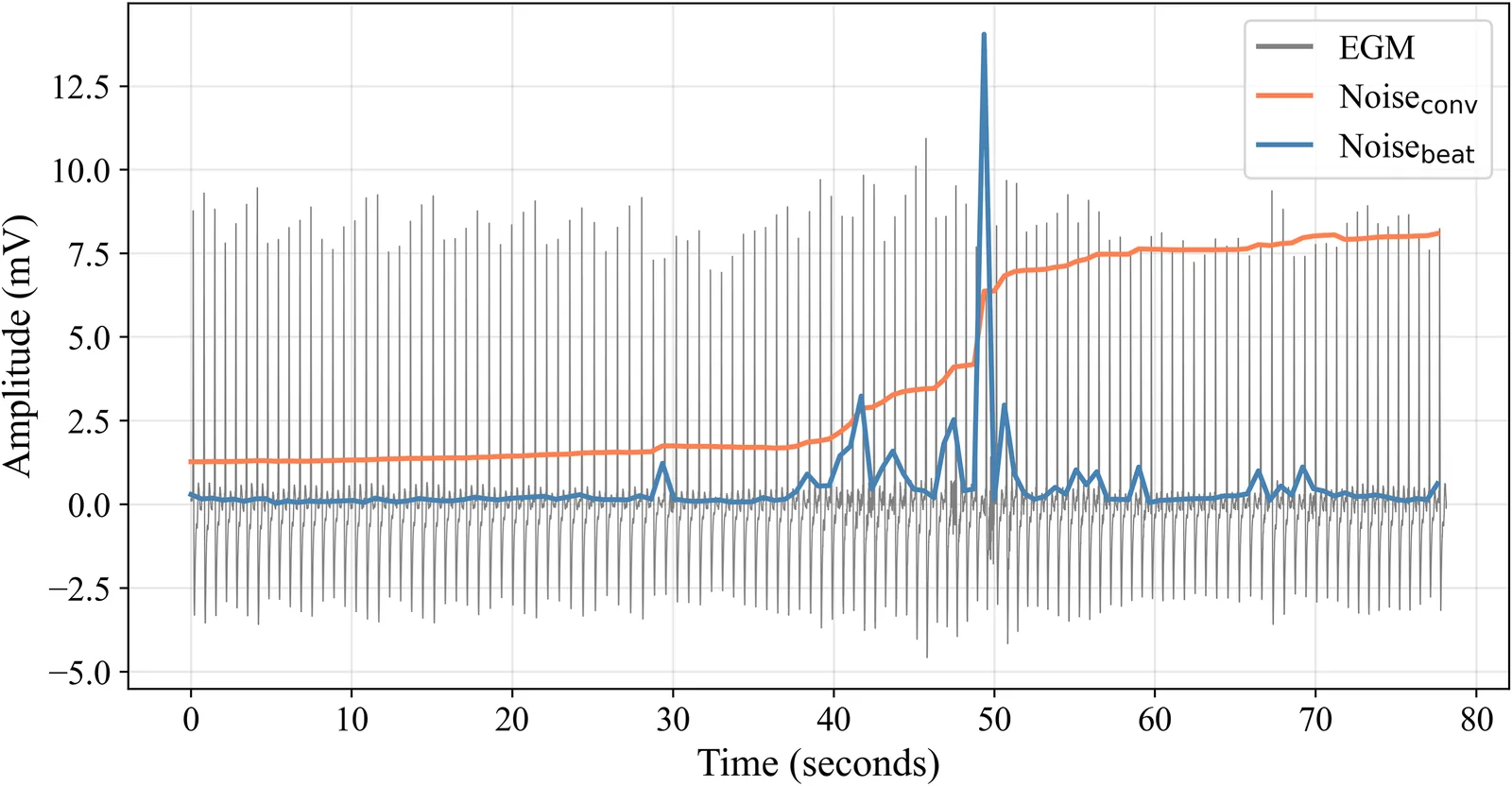
Visualization example of EGM signal (*gray*) alongside noise estimates per beat (*blue*) and the corresponding convolved noise signal (*orange*). EGM = electrogram.

An example of the noise signals at different stages of the process, displayed alongside the corresponding EGM signal, is presented in Figure 4. As shown, the convolved noise provides a smoothed noise profile, effectively accounting for noise effects across beats and enabling clearer identification of sustained noise trends that may affect STV calculations.

### STV measurement

QT intervals were automatically measured using a previously validated algorithm by Smoczyńska et al.^22^ To account for heart rate variations, QT intervals were corrected using Bazett’s formula by dividing the QT interval by the square root of the R-wave to R-wave interval, as expressed in the following equation:

(3) $${QT}_{c}=\frac{QT}{\sqrt{RRI}}$$

After this correction, STV was computed over 31 consecutive beats using the standard STV formulation^12^:

(4) $$STV=\frac{\sum_{i=1}^{31}\left|{QTc}_{n+1}-{QTc}_{n}\right|}{30\;\sqrt{2}}$$

STV values were calculated for all transmissions. In cases of episodes associated with arrhythmic events, STV measurements were restricted to segments of the recording that did not contain ventricular arrhythmias.

To ensure the accuracy of STV measurements, corrected QT (QTc) intervals associated with ABs were removed given that these beats exhibit distinct repolarization characteristics that could distort STV calculations. To further minimize potential artifacts, the QTc interval immediately preceding an AB and the 2 intervals after it were also discarded.

In addition, QTc intervals affected by excessive noise were also removed from the analysis. Noise levels were quantified using the automatic algorithm described in the section Noise-detection algorithm, with a threshold value set at 850 units. This threshold was determined based on visual inspection of recordings exhibiting noise.

A sensitivity analysis using Fridericia’s correction was also performed to assess the robustness of the results to the choice of QT correction method (see Supplemental Material for details).

Finally, STV values before and after AB exclusion, as well as before and after noise filtering, were compared using the paired Wilcoxon signed-rank test to assess the potential confounding effects of these factors on STV values.

### STV comparison

#### STV paired comparison

As outlined in the section Database, the database consists of 2 distinct patient groups:

• Group 1: patients with only baseline recordings and no recorded arrhythmic episodes.
• Group 2: patients with both baseline recordings and recordings associated with arrhythmic episodes.

Based on these 2 groups, the following STV comparisons were conducted:

• Group 2 baseline vs group 2 arrhythmic events: this paired comparison aims to validate existing evidence suggesting an increase in STV values before arrhythmic events. By analyzing changes within the same individuals, this comparison assesses whether STV levels rise significantly before arrhythmic episodes, potentially serving as an early marker of arrhythmic risk. By analyzing recordings spanning approximately 140 seconds before the arrhythmic event, this comparison evaluates whether the STV values are elevated at least 2 minutes before the event, supporting its potential as an early marker for arrhythmic risk.
• Group 1 baseline vs group 2 arrhythmic events: this unpaired comparison evaluates differences in absolute STV values between the 2 groups. The goal is to assess whether STV values during arrhythmic episodes in group 2 significantly differ from baseline values in patients without arrhythmic events (group 1). This analysis provides insights into the magnitude of STV changes associated with arrhythmias, independent of individual patient baselines.
• Group 1 baseline vs group 2 baseline: the objective of this comparison is to determine whether patients with higher baseline STV values are at an elevated risk of developing arrhythmic events. By comparing baseline recordings between patients who experienced arrhythmias (group 2) and those who did not (group 1), this analysis explores the potential of baseline STV as a predictor of future arrhythmic risk.
• Group 1 baseline vs group 1 baseline subsequent transmission: this comparison serves as a negative control. It examines patients in group 1 who had no arrhythmic events but multiple transmissions. STV values are compared between 2 different time points to ensure that any observed changes in other comparisons are not caused by unrelated temporal variability.

For all comparisons, results were reported as median values with interquartile ranges (IQRs). To determine the statistical significance of differences in STV measurements across the defined patient groups and conditions, appropriate nonparametric statistical tests were used. A paired Wilcoxon signed-rank test was used to compare STV values between baseline and arrhythmic events within group 2, assessing intrapatient changes. For comparisons involving independent samples (ie, between groups 1 and 2), the Mann–Whitney U test was applied. *P* ≤ .05 was considered statistically significant.

#### STV trend comparison

To further characterize the temporal evolution of STV before arrhythmic events, we evaluated the trajectory of STV over time across the study groups.

For group 2, STV trajectories up to 120 seconds preceding the arrhythmic event were analyzed. The portion of the EGM containing the arrhythmic episode was excluded. For group 1, corresponding time windows were selected from baseline recordings to provide a reference.

Mean STV trajectories were computed across patients for each group and are presented to illustrate temporal patterns. To preserve clarity, variability bands are not displayed; however, interpatient variability is accounted for through statistical comparisons performed at the patient level.

To quantitatively compare STV trajectories, the area under the curve (AUC) was computed for each patient over the analyzed time window. This approach provides a summary measure of the overall STV values over time while preserving patient-level variability. Group differences in AUC values were assessed using the Mann–Whitney U test. *P* ≤ .05 was considered statistically significant.

## Results

### STV confounding by ABs and noise

**Figure 5:**
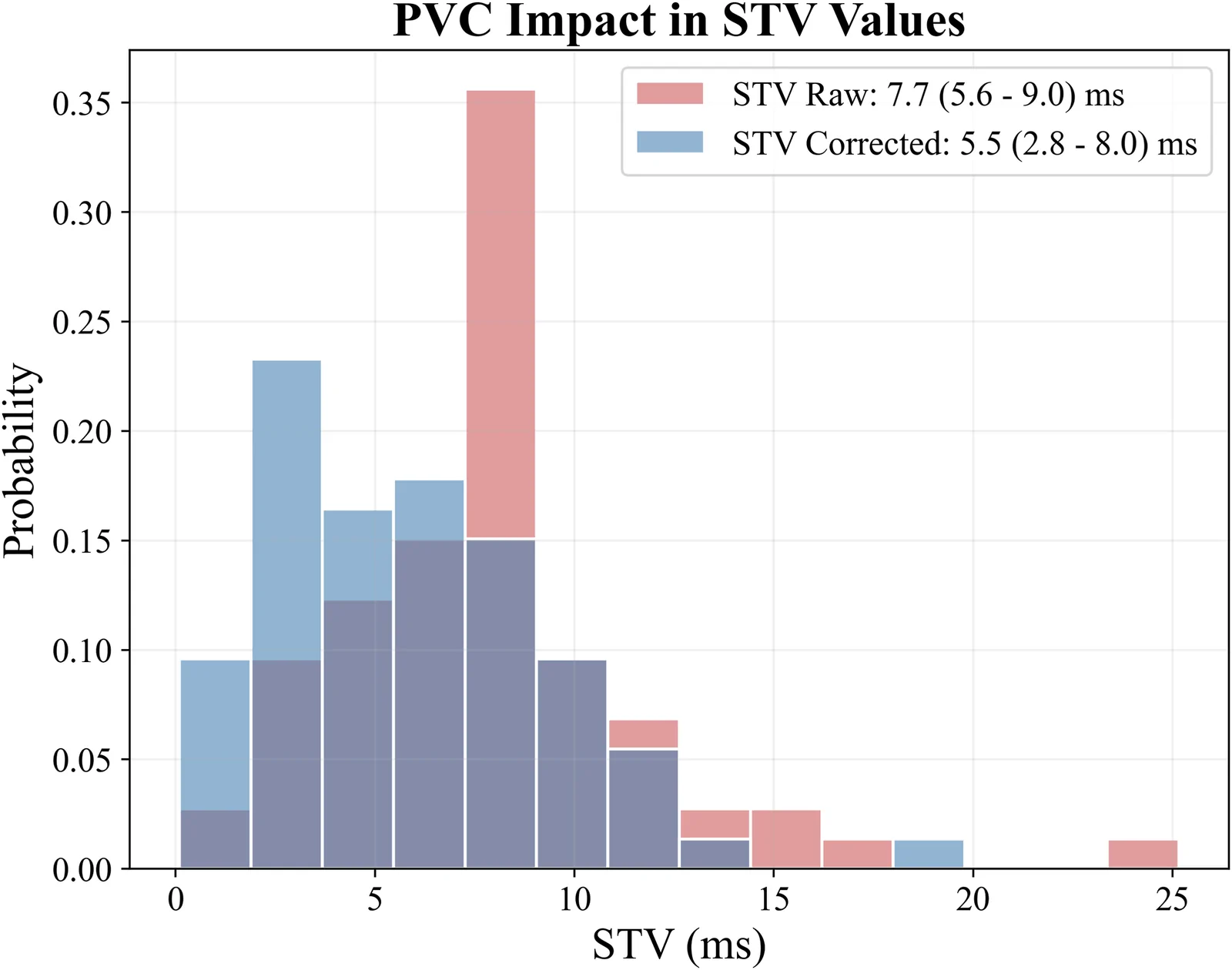
STV values before (*red*) and after (*blue*) abnormal beat correction. The legend presents the median and interquartile range for both conditions. PVC = premature ventricular contraction; STV = short-term variability.

Our findings suggest that ABs significantly increase STV values. The STV raw values (red) and STV corrected values (blue) after removing ABs are presented in Figure 5. The median and IQR were 7.7 ms (5.6–9.0) for the STV before and 5.5 ms (2.8–8.0) after correction. Differences in STV value were significant with *P* < .00001. The median change in STV per AB was 0.4 ms (0.2−0.6).

**Figure 6:**
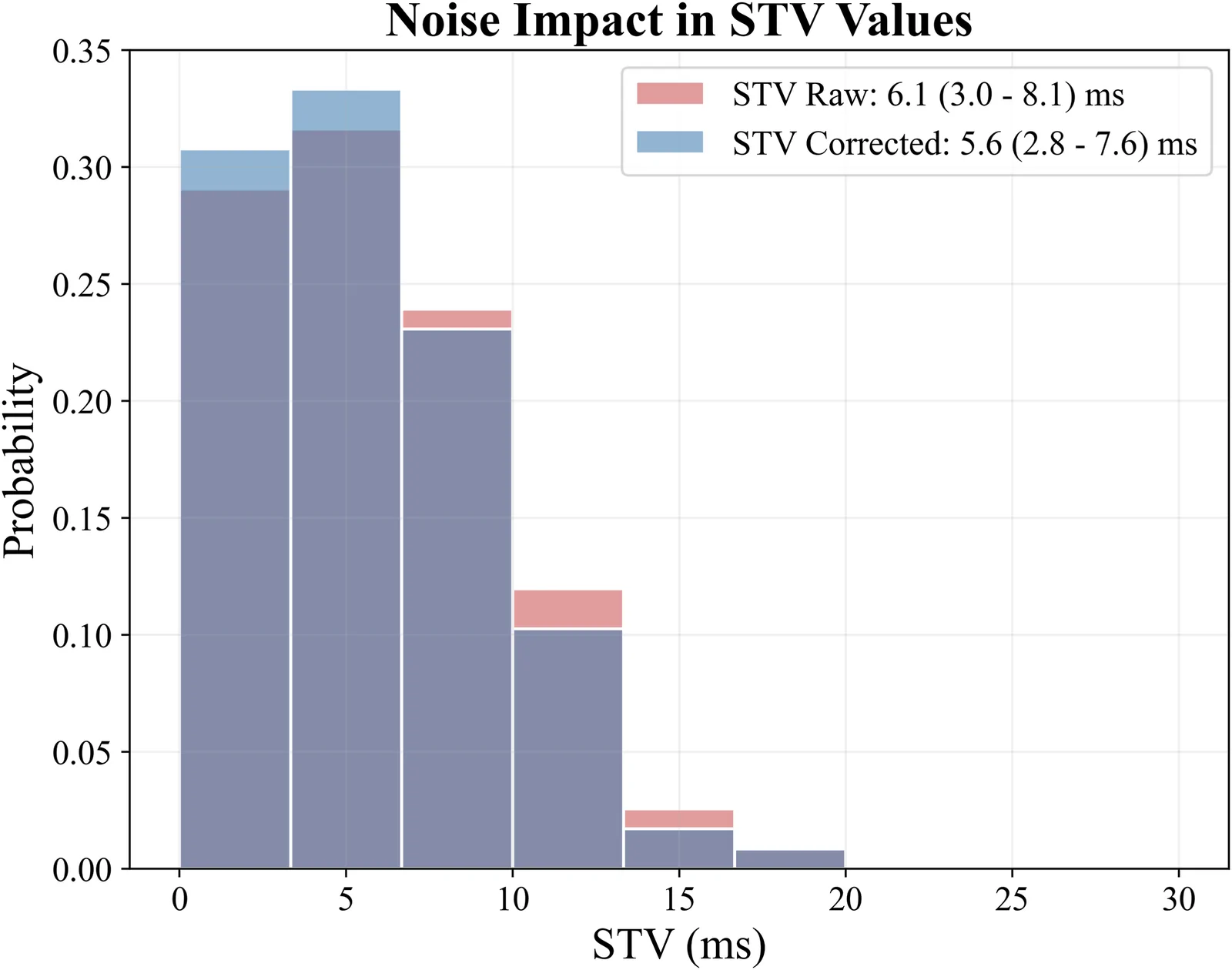
STV values before (*red*) and after (*blue*) noise correction. The legend presents the median and interquartile range for both conditions. STV = short-term variability.

In addition to ABs, our analysis demonstrates that STV values are also influenced by noise. The STV values of the patients in the analysis before (red) and after removing noisy beats (blue) are presented in Figure 6. The median and IQR for both conditions were 6.1 ms (3.0–8.1) for the STV before and 5.6 ms (2.8–7.6) after correction. Differences in STV values were significant with *P* < .00017. It is noteworthy that this comparison includes all patients, regardless of the presence of noise, which may have attenuated the observed differences.

### STV comparisons among groups

**Figure 7:**
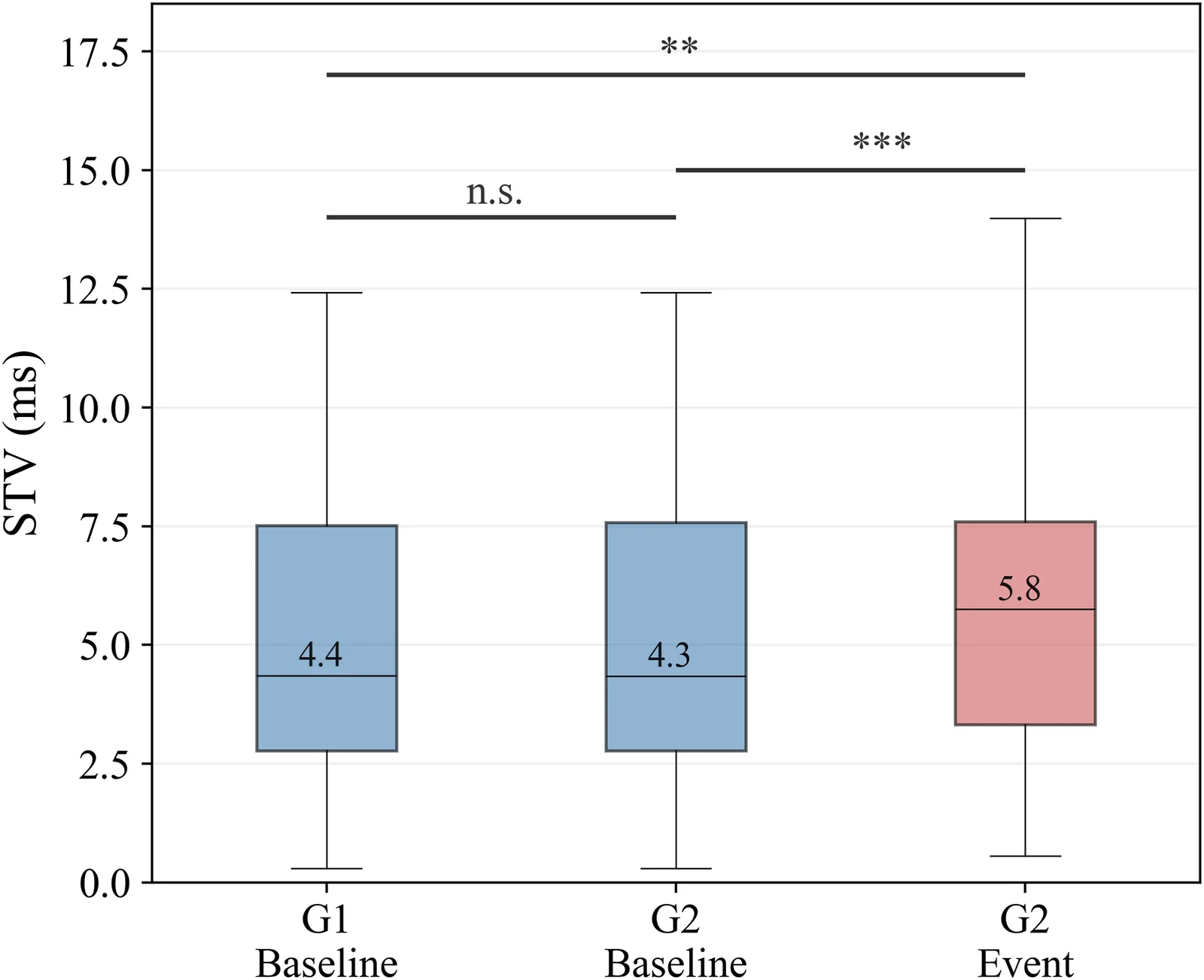
Comparison of STV values (excluding values associated with noisy beats) between baseline recordings of G1 baseline and G2 baseline, and arrhythmic event recordings for G2 event. The figure illustrates differences in STV values across groups and conditions, highlighting changes associated with arrhythmic events in G2. Statistical significance is indicated as follows: n.s. *P* > .05; ∗∗*P* ≤ .01; ∗∗∗*P* ≤ .001. G1 = group 1; G2 = group 2; n.s. = not significant; STV = short-term variability.

The comparison of STV values after filtering abnormal and noisy beats across the different patient groups is presented in Figure 7 and Table 1. STV values were significantly higher in recordings preceding arrhythmic events than in baseline measurements. No significant differences were observed between baseline STV values of groups 1 and 2. These results were consistent when using Fridericia’s correction, with slightly lower absolute STV values but no change in the statistical significance of group differences (see Supplemental Material for details).

**Table 1:** Comparison of STV values after exclusion of noisy and abnormal beats across BL1, BL2, and arrhythmic event recordings (VT/VF) for group 2 (event)

Comparison	STV condition A	STV condition B	*P* value
BL2 vs event	4.3 (2.8–7.6)	5.8 (3.3–7.6)	<.0001
BL1 time 0 vs BL1 time 1	4.4 (2.8–7.5)	4.4 (2.7–7.3)	.1961
BL1 vs BL2	4.4 (2.8–7.5)	4.3 (2.8–7.6)	.9418
BL1 vs event	4.4 (2.8–7.5)	5.8 (3.3–7.6)	.0018

In addition, STV values are compared between 2 different time points (time 0 and time 1) for the same patients in group 1. Results are presented as median values with interquartile ranges. *P* values correspond to statistical comparisons between groups.

BL1 = baseline recordings from group 1; BL2 = baseline recordings from group 2; STV = short-term variability; VF = ventricular fibrillation; VT = ventricular tachycardia.

**Figure 8:**
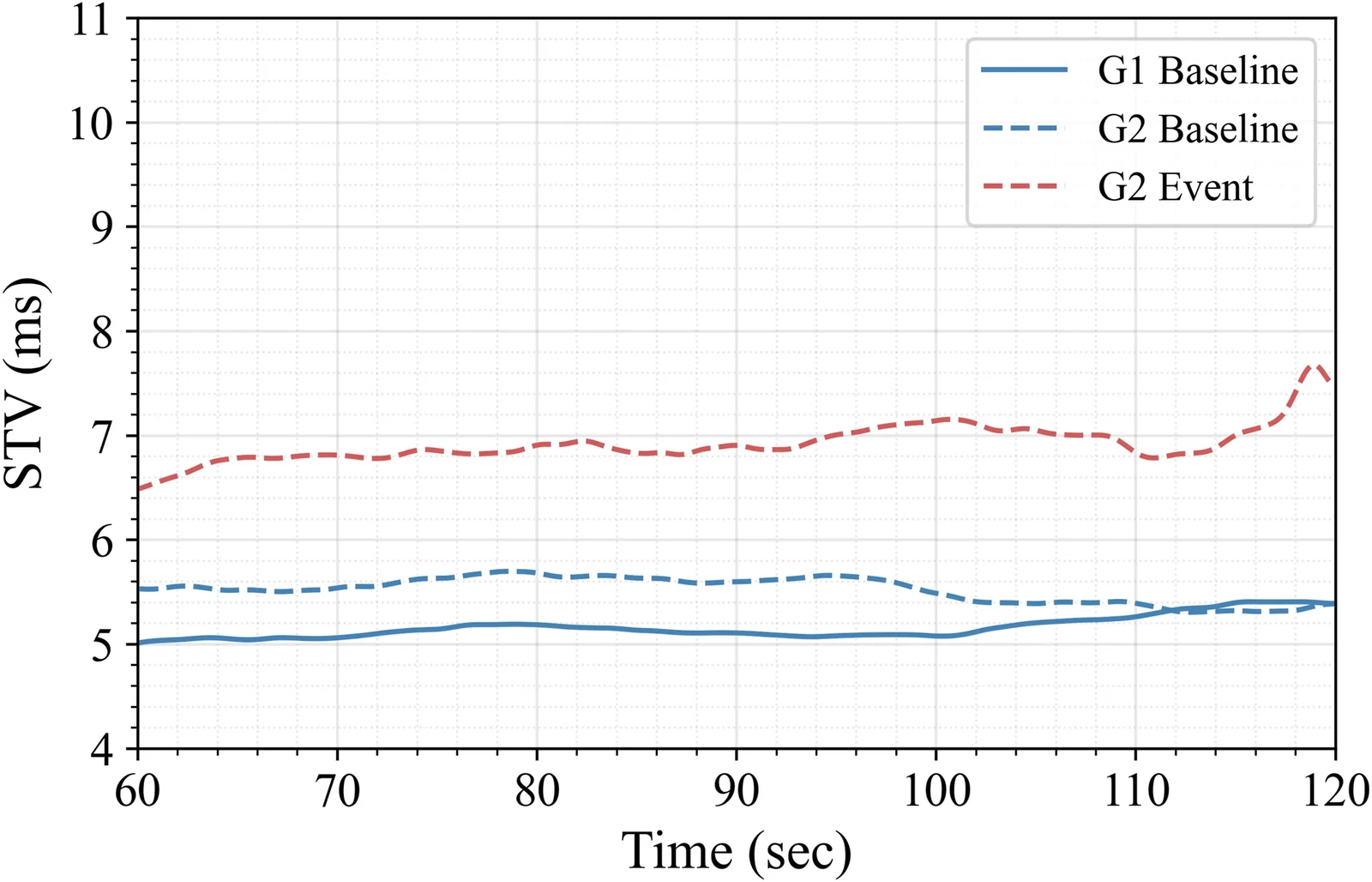
Comparison of longitudinal STV trajectories across study groups. Mean STV values are shown for baseline recordings in G1 baseline, baseline recordings in G2 baseline, and recordings preceding arrhythmic events in G2 event. Trajectories represent STV evolution over time before the event; the arrhythmic episode itself is excluded. G1 = group 1; G2 = group 2; STV = short-term variability.

The comparison of STV trajectories after filtering abnormal and noisy beats across the study groups is presented in Figure 8 and Table 2. STV trajectories in recordings preceding arrhythmic events were higher than those observed in baseline recordings from both groups 1 and 2. Quantitative comparisons based on AUC values are presented in Table 2.

**Table 2:** Comparison of area under the curve of STV trajectories across study groups, including BL1, BL2, and recordings preceding arrhythmic events in group 2 (event)

Comparison	STV condition A	STV condition B	*P* value
BL2 vs event	244 (169–443)	397 (221–528)	.0003
BL1 vs event	237 (135–400)	397 (221–528)	<.0001
BL1 vs BL2	237 (135–400)	244 (169–443)	.1980

Results are presented as median values with interquartile ranges. *P* values correspond to statistical comparisons between groups.

BL1 = baseline recordings from group 1; BL2 = baseline recordings from group 2; STV = short-term variability.

**Figure 9:**
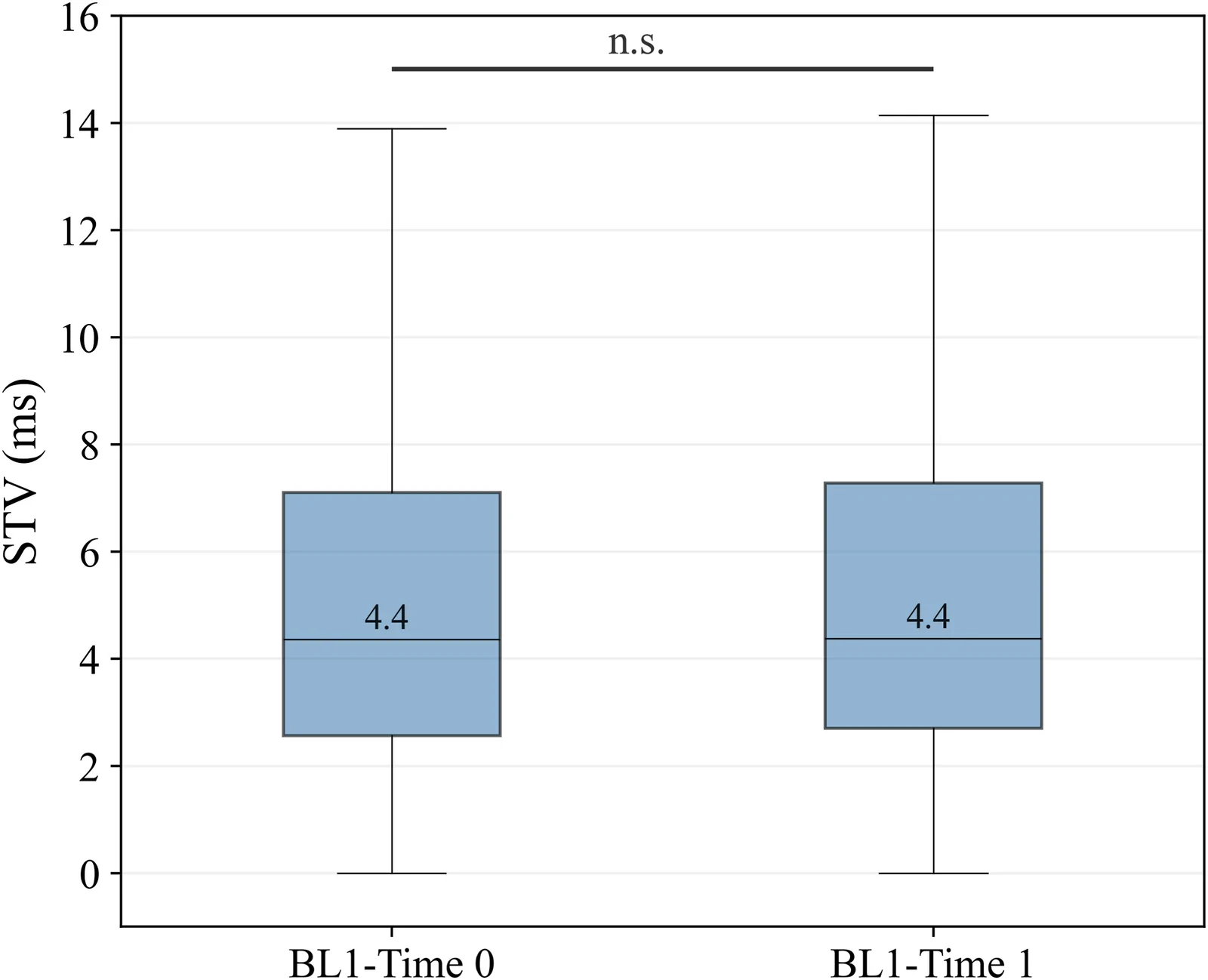
Negative control comparison for patients in group 1. STV values are compared between 2 different time points: “Time 0” representing an initial time point and “Time 1” representing a subsequent time point. The mean interval between “Time 0” and “Time 1” was 94.7 ± 76.6 days. Statistical significance is indicated as follows: n.s. *P* > .05; ∗∗*P* ≤ .01; ∗∗∗*P* ≤ .001. BL1 = baseline recordings from group 1; BL2 = baseline recordings from group 2; n.s. = not significant; STV = short-term variability.

In addition, within-group comparison in group 1 between 2 different time points (time 0 and time 1) is presented in Table 1 and Figure 9. This comparison serves as a negative control, confirming the stability of baseline STV values in patients without arrhythmic events. The mean time separation between time points 0 and 1 was 95 ± 77 days.

**Figure 10:**
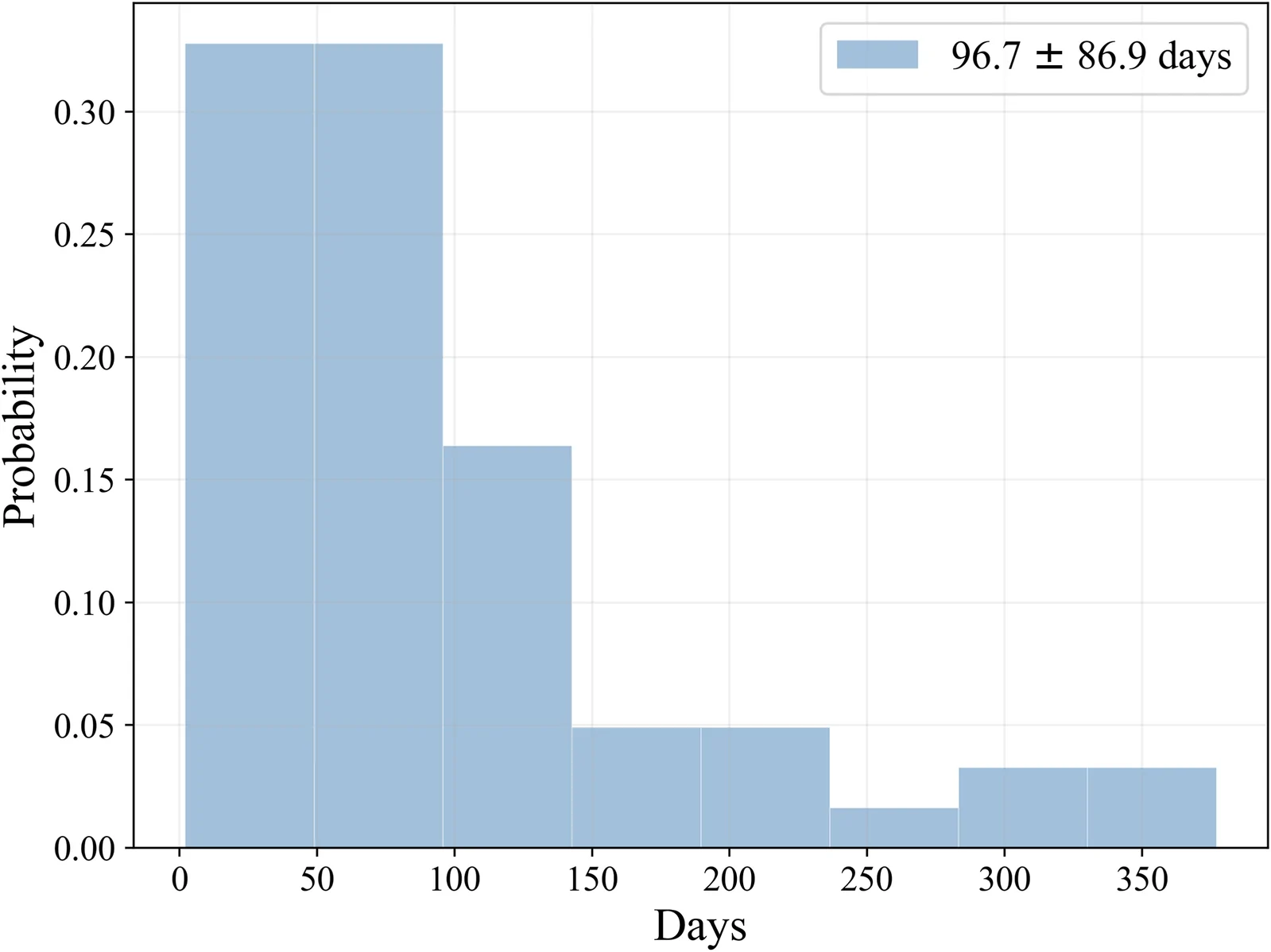
Time separation between baseline and arrhythmic recordings for patients having cardiac events (group 2).

The time horizon over which changes in STV occur is critical for evaluating its predictive utility. The distribution of time intervals between baseline recordings and the occurrence of arrhythmic events in patients from group 2 is presented in Figure 10. The analysis reveals that the mean time separation between baseline assessments and arrhythmic episodes is 97 ± 87 days, indicating substantial variability among patients. The IQR spans from 31 to 105 days, suggesting that 75% of the events occurred within 105 days after previous interrogation.

## Discussion

This study aimed to evaluate the potential of STV for the prediction of arrhythmic events in CRT-D and ICD patients. By leveraging anonymized data from Medtronic CRT-D and ICD devices, we investigated STV changes in 2 patient groups: those with only baseline recordings (group 1) and those with both baseline and arrhythmic event recordings (group 2). Only MVT and PVT/VF arrhythmic events were considered, whereas other arrhythmias were excluded from the analysis.

The findings of our study contribute to the growing body of evidence supporting STV as a marker for continuous arrhythmic risk assessment while also highlighting its limitations in long-term risk stratification.

### STV for prediction of arrhythmic events

Our analysis revealed a significant increase in STV values in recordings preceding arrhythmic events compared with baseline recordings in group 2. Notably, recordings provided approximately 140 seconds of usable data for STV measurement, which suggests that STV increases are detectable within at least 140 seconds before an arrhythmic event. As observed in Table 1 (first row), the median STV value before the arrhythmic event was 5.8 ms, whereas it was 4.3 ms at baseline (*P* < .0001). This observation aligns with previous preclinical and clinical studies suggesting that elevated STV indicates heightened repolarization dispersion and electrical instability. However, the observed mean time interval between baseline recordings and arrhythmic events was 96.7 ± 86.9 days, and therefore, it remains unclear when the STV increase occurs relative to the arrhythmic event.

The analysis of STV trajectories showed that STV values in recordings preceding arrhythmic events were significantly higher than those observed in the corresponding baseline recordings (Table 2). As illustrated in Figure 8, STV values remain elevated throughout the analyzed pre-event window, with a tendency toward a gradual increase closer to the event. This analysis suggests that STV trajectories separate primarily according to recording condition (baseline vs pre-event). Baseline recordings from both groups show similar trajectory patterns, whereas recordings preceding arrhythmic events consistently exhibit higher STV levels. However, as mentioned earlier, given that the recordings include a limited pre-event duration, it is not possible to determine when the initial increase in STV occurs relative to the arrhythmic event.

In addition, the within-group comparison in group 1 between 2 different time points (time 0 and time 1) confirmed the stability of baseline STV values in patients without arrhythmic events (Table 1 and Figure 9). This lack of significant difference serves as a negative control and reinforces the specificity of STV changes preceding arrhythmic events.

These findings suggest the potential for integrating STV monitoring into CRT-D and ICD algorithms for the prediction of ventricular arrhythmias, although further investigation is necessary to determine how early such changes can reliably indicate an elevated risk of arrhythmias.

### STV for risk stratification

Despite the encouraging indications of STV as a predictive marker (at least up to 2 minutes before the arrhythmic event), our findings reveal that baseline STV values alone are not enough for reliable long-term risk stratification. The absence of significant differences in baseline STV values between groups 1 and 2 suggests that baseline measurements, in isolation, may not effectively differentiate patients who will experience arrhythmic events from those who will not over extended follow-up periods. As shown in Table 1 (second row), the median baseline STV value for patients without arrhythmic events is 4.4 ms compared with 4.3 ms for patients who subsequently experienced arrhythmic events (*P* = .9418) after the baseline recording. These results imply that although elevated STV levels may signal near-term risk of arrhythmias, baseline STV levels alone might not capture the complex, multifactorial nature of long-term arrhythmic risk.

Furthermore, an exploratory analysis of longitudinal baseline STV trajectories across multiple transmissions showed largely overlapping temporal patterns between patients with and without subsequent arrhythmic events, with no additional discriminatory value beyond single baseline measurements (see Supplemental Material for details).

Given that our data cover only a 1-year period, it is also possible that some patients classified as event-free in this analysis may develop arrhythmic events in the future, highlighting the need for caution in interpretation. Further research is required to validate these findings and to explore whether longitudinal STV trends or additional risk factors could enhance predictive accuracy.

### Confounding of noise and ABs on STV measurements

One of the primary challenges in using STV for continuous arrhythmic risk monitoring is its sensitivity to noise and ABs. To mitigate this issue, our methodology excluded QTc intervals associated with ABs and periods of excessive noise to ensure the reliability of STV measurements.

As shown in Figure 5, the presence of ABs significantly increases STV values. The median increase in STV per AB was calculated as 0.4 ms (0.2–0.6), demonstrating the impact of ABs on STV calculations.

**Figure 11:**
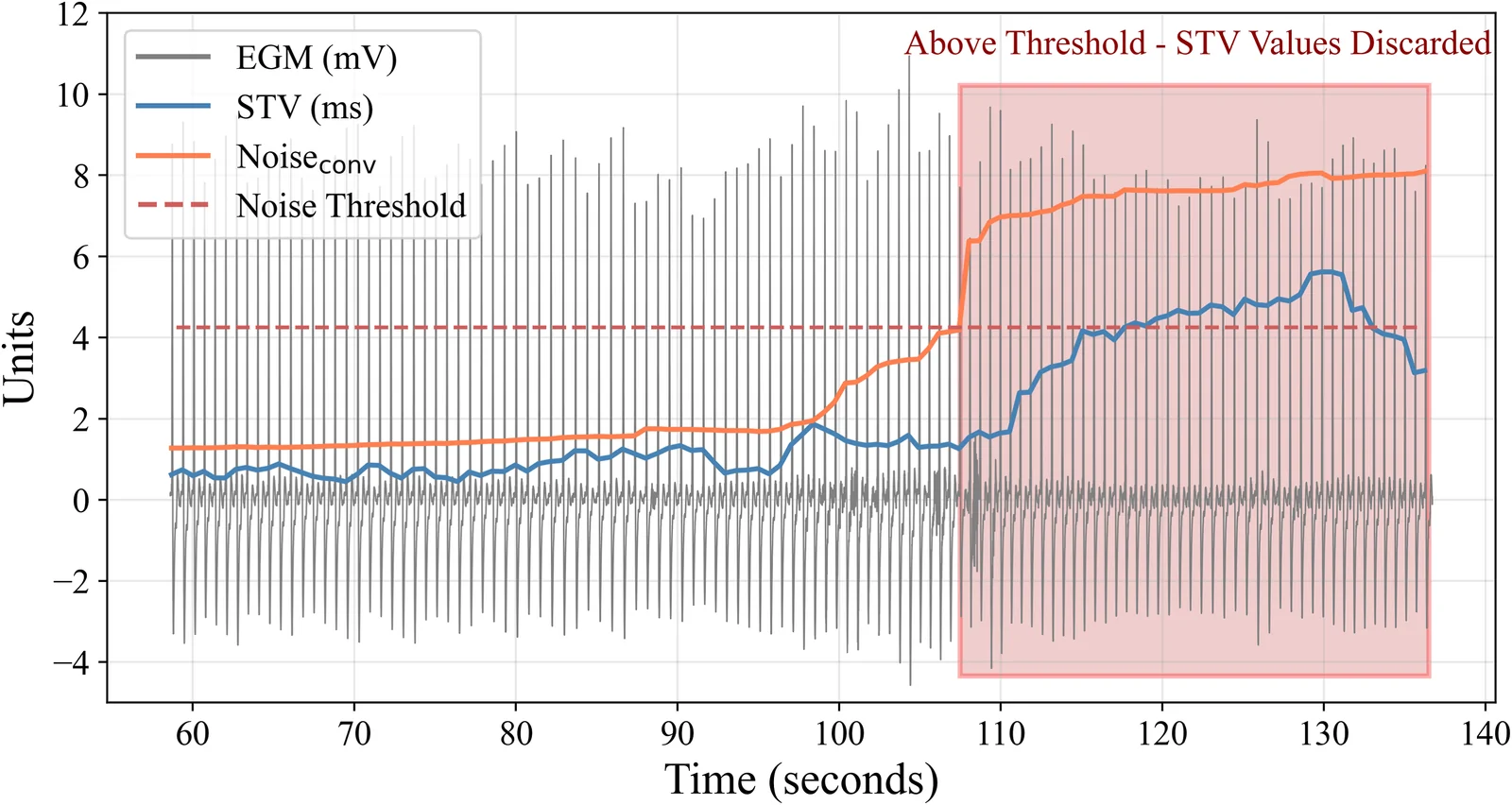
Example of a recording illustrating the impact of noise on STV values. As noise levels increase, a corresponding rise in STV values is observed, highlighting the potential confounding effect of noise on STV measurements. EGM = electrogram; STV = short-term variability.

A similar effect was observed in the case of noise, which also led to a significant increase in STV values. The median change in STV owing to noise was 0.5 ms, reinforcing the confounding influence of signal disturbances on STV assessments. This phenomenon, where STV values (blue line) increase in response to rising noise levels (orange line), is presented in Figure 11. It is noteworthy that this comparison included all patients, regardless of the presence of noise, which may have attenuated the observed differences. To minimize the confounding effect of noise, QTc intervals corresponding to beats where noise exceeded a predefined threshold (850 units for our EGM recordings) were excluded from the STV calculation. In the example depicted in Figure 11, STV values within the red box were identified as unreliable and, therefore, automatically discarded in the calculation of STV.

Given that abnormal and noisy beats increase STV values, their inclusion could confound the results of our analysis. Therefore, we systematically excluded those beats from the STV calculation. In the case of ABs, to prevent any residual influence, the beat preceding an AB and the 2 subsequent beats were removed from the analysis. In addition, noisy beats were automatically identified and excluded using the noise-detection algorithm detailed in the section Noise-detection algorithm.

### Temporal relationship between baseline and arrhythmic events

The observed mean time interval of 96.7 ± 86.9 days between baseline recordings and arrhythmic events in group 2 highlights significant temporal variability in arrhythmic risk among patients. Therefore, our analysis does not indicate when the observed STV increase occurs within this period, raising questions about the timing and frequency of optimal monitoring. These findings underscore the need for prospective studies with more frequent sampling and larger patient cohorts to establish clinically relevant time frames for STV-based risk prediction.

Despite this limitation, elevated STV values were observed within the available pre-event recording window, approximately 140 seconds before the arrhythmic event, underscoring its potential as a short-term predictive marker.

Furthermore, a sensitivity analysis was performed by progressively restricting the time interval between baseline STV measurements and arrhythmic events using increasing temporal thresholds. For each threshold, within-patient differences (ΔSTV = STV_event_ − STV_baseline_) were computed and analyzed using the Wilcoxon signed-rank test. As the temporal window increased, the sample size also increased, enabling more robust comparisons. The median ΔSTV values stabilized across thresholds of ≥60–90 days, remaining consistently positive (approximately 0.7 to 0.9 ms) and indicating higher STV values at the time of arrhythmic events than baseline. These findings suggest that the observed increase in STV is robust and not driven by the specific timing between baseline and event recordings. The results of this sensitivity analysis are described in Section 1 of the Supplemental Material.

### Clinical implications and future directions

The integration of STV monitoring into CRT-D and ICD devices holds significant clinical promise. Tracking STV dynamics could facilitate early detection of arrhythmic episodes, potentially enabling timely therapeutic interventions that prevent device shocks and SCD.

Translation of these findings into clinical practice requires addressing several technical and methodological challenges. The signal preprocessing and noise reduction methods used in this study were designed for offline analysis of telemetry EGMs and are not part of current device-based signal processing pipelines used for sensing and therapy delivery. In addition, the implementation of STV monitoring would introduce additional computational requirements, which could affect device resources such as processing load and power consumption. These constraints may be mitigated by optimized implementations, for example, by performing STV analysis intermittently (eg, at predefined intervals or under specific physiological conditions) rather than continuously. Consequently, further work is needed to evaluate the feasibility of real-time STV monitoring in implantable devices, including the impact of signal quality, sampling characteristics, and computational constraints, as well as the trade-offs between predictive performance and device resource utilization.

An additional consideration for clinical translation is the temporal relationship between STV changes and arrhythmic events. In this study, elevated STV values were observed within the available pre-event window (∼120 seconds). Notably, STV values were already significantly higher than baseline at the beginning of this interval, suggesting that the primary increase in STV occurs earlier than the portion of the signal captured in the recordings. Therefore, the present data likely reflect a late phase of STV elevation rather than its onset. Although the precise timing of this increase cannot be determined, these findings suggest that real-time STV monitoring could potentially detect earlier changes than those observed here, thereby providing a longer window for intervention. In principle, such changes could be used to trigger preventive strategies, including adaptive pacing or other device-based interventions, although their effectiveness in this setting remains to be established. However, a key limitation of the present study is the lack of continuous pre-event recordings, which prevents full characterization of the temporal evolution of STV before arrhythmic events. Future studies incorporating continuous monitoring will be required to determine the timing and magnitude of STV changes and to assess whether these can be leveraged for real-time clinical decision making.

In addition, translation into clinical practice will require a better understanding of the physiological factors influencing STV variability across diverse patient populations, which may be essential for tailoring individualized risk prediction models. Furthermore, although survival analysis represents a standard approach for evaluating long-term prognostic markers, its application in the present study is limited by the lack of detailed demographic and clinical covariates, which precludes appropriate adjustment for potential confounders. In addition, baseline STV values did not differ between patients with and without subsequent arrhythmic events, suggesting limited prognostic value of baseline STV for long-term risk stratification in this dataset. Future studies incorporating comprehensive clinical information and continuous longitudinal monitoring will be necessary to assess whether STV can contribute to multivariable time-to-event models and improve long-term risk prediction.

Finally, future research should explore the integration of STV with complementary predictive markers such as heart rate variability and autonomic tone indices to improve risk stratification. In addition, clinical trials evaluating the efficacy of STV-guided interventions, such as preemptive pacing strategies, will be critical in demonstrating the practical utility of STV monitoring in reducing arrhythmic event rates and improving patient outcomes.

In summary, our findings support the role of STV as a marker for the prediction of ventricular arrhythmias in the CRT-D and ICD population. Although a significant elevation of STV values was observed within ∼2 minutes of arrhythmic events, the timing of this increase relative to event onset remains unclear. Consequently, although STV holds potential for risk assessment and intervention planning, additional research is necessary to refine temporal interpretations and improve its clinical applicability. Future studies should aim to validate STV-based predictive models and assess their efficacy in large-scale clinical trials, ultimately contributing to improved patient management and reduced incidence of SCD.

## Conclusion

In this study, we evaluated the potential of STV as a predictor of ventricular arrhythmias in patients with CRT-D and ICD devices and its applicability in real-world monitoring during daily living. Our findings support the role of STV as a short-term marker for arrhythmic risk, as evidenced by a significant elevation in STV values preceding arrhythmic events compared with baseline recordings.

Furthermore, our study provides insights into the confounding effects of AB depolarizations and noise on STV measurements. We demonstrate that both factors can inflate STV values, potentially leading to misinterpretation of arrhythmic risk. To address this, we propose methodological refinements, including the exclusion of confounded QTc intervals and the implementation of robust noise-detection algorithms, to enhance the accuracy of STV-based risk assessment.

By addressing these challenges, our work contributes to the advancement of STV as a potential clinical tool for arrhythmic risk prediction and patient management. Future research should focus on refining STV measurement techniques, optimizing predictive models, and validating these findings in larger clinical cohorts to further establish STV’s role in real-world applications.

## Disclosures

Alfonso Aranda Hernández is employed by Medtronic. Mathias Meine has no conflicts to disclose. Marc Vos reports previous research funding from multiple organizations including Medtronic.

## Footnotes

Appendix Supplementary data associated with this article can be found in the online version at https://doi.org/10.1016/j.hroo.2026.06.005.
