# Supplementary material for: Automatic measurement of short-term variability of repolarization on human intracardiac electrograms predicts VT/VF events
Source: Heart Rhythm O2. 2026 Jun 16;7(9):1762–74. doi: 10.1016/j.hroo.2026.06.005 (PMC13594440; doi:10.1016/j.hroo.2026.06.005)
Supplement: Supplementary file 1 [file mmc1.docx]

Supplementary Material

# 1. ****Impact of Temporal Proximity Between Baseline STV and Arrhythmic Events****

To assess the potential influence of the time interval between baseline STV measurements and arrhythmic events, we performed a sensitivity analysis evaluating how this temporal proximity affects the observed differences in STV.

Figure S1 illustrates the distribution of time intervals between baseline recordings and corresponding ventricular arrhythmic events. As shown, only a limited number of cases are available within very short time intervals (e.g., < 7 days or < 14 days), which precludes meaningful statistical analysis within these narrow windows due to insufficient sample size.

To address this limitation, we performed a sensitivity analysis by progressively restricting the maximum allowable time difference between baseline STV measurements and arrhythmic events using increasing temporal thresholds. Specifically, thresholds ranging from 30 to 120 days were evaluated.

For each threshold, only patients with a baseline recording occurring within the specified time window prior to the arrhythmic event were included. For these patients, within-subject differences in STV were computed as:

$$\Delta STV=STV_{\text{event}}-STV_{\text{baseline}}$$

These paired differences were then analyzed using the Wilcoxon signed-rank test.

## ****Results****

As summarized in Table S1, the number of available patient pairs increased with wider temporal thresholds, ranging from 17 patients at 30 days to 78 patients at 360 days.

At the shortest evaluated window (30 days), the median ΔSTV was -0.2(-1.9–1.0) ms, reflecting high variability and limited statistical power. However, as the temporal window increased, the median ΔSTV values became consistently positive and stabilized.

For thresholds ≥ 60–90 days, the median ΔSTV ranged between approximately 0.7 and 0.9 ms, with relatively stable interquartile ranges despite increasing sample size. Across all available patient pairs, ΔSTV was significantly greater than zero (Wilcoxon signed-rank test, p = 0.0005).

## ****Interpretation****

These results indicate that STV values at the time of arrhythmic events are consistently higher than baseline values, and that this difference is robust across a wide range of temporal intervals between baseline and event recordings.

Importantly, the stabilization of ΔSTV across increasing temporal thresholds suggests that the observed increase in STV is not strongly dependent on the exact timing of the baseline measurement relative to the arrhythmic event. However, due to the limited number of cases with very short time intervals between baseline recordings and events, it is not possible to determine whether STV changes become more pronounced in close temporal proximity (e.g., within days) to the arrhythmic event.

Future studies with higher temporal resolution or continuous monitoring closer to arrhythmic events would be required to further characterize the short-term dynamics of STV.

Figure S1. Cumulative number of patients with arrhythmic events as a function of the time interval (up to 120 days) from baseline recording to event occurrence. The plot shows that only a small number of events occurred within the first days after baseline, limiting the feasibility of subgroup analyses in very short temporal windows.

| Threshold (days) | N (patients) | ΔSTV Median (IQR) |
| --- | --- | --- |
| 30 | 17 | -0.2 (-1.9–1.0) |
| 60 | 31 | 0.5 (-0.7–2.4) |
| 90 | 44 | 0.7 (-0.6–2.3) |
| 120 | 62 | 0.7 (-0.6–2.4) |
| 150 | 64 | 0.7 (-0.6–2.3) |
| 180 | 66 | 0.7 (-0.6–2.4) |
| 210 | 71 | 0.8 (-0.6–2.5) |
| 240 | 74 | 0.8 (-0.6–2.7) |
| 270 | 75 | 0.9 (-0.6–3.0) |
| 300 | 76 | 0.9 (-0.6–3.2) |
| 330 | 77 | 0.9 (-0.5–3.1) |
| 360 | 78 | 0.9 (-0.5–3.1) |

Table S1. Sensitivity analysis of STV differences as a function of the time interval between baseline recordings and ventricular arrhythmic events. For each temporal threshold (in days), only patients with baseline recordings within the specified interval prior to the event were included. Within-patient differences (ΔSTV = STV_event_ - STV_baseline_) are reported as median (IQR). The analysis is limited to a maximum interval of one year.

# 2. ****QT Fridericia Correction****

To assess the robustness of the STV findings to the choice of QT correction method, we repeated the analysis using Fridericia’s correction (QTcF) instead of Bazett’s correction (QTcB). Given that Bazett’s formula is known to overcorrect at higher heart rates, this analysis evaluates whether the observed differences in STV are consistent under an alternative correction approach.

Figure S2 shows the distribution of STV values calculated using Fridericia’s correction, excluding noisy and abnormal beats, across baseline recordings from Group 1 (G1 Baseline), baseline recordings from Group 2 (G2 Baseline), and arrhythmic event recordings from Group 2 (G2 Event).

Consistent with the main analysis, STV values were significantly higher in recordings associated with arrhythmic events compared to baseline recordings. No significant differences were observed between baseline recordings of Group 1 and Group 2.

Statistical comparisons are summarized in Table S2. Paired comparisons within Group 2 (baseline vs event) were performed using the Wilcoxon signed-rank test, while independent comparisons between groups were performed using the Mann–Whitney U test. Results are reported as median values with interquartile ranges (IQR), and a p-value ≤ 0.05 was considered statistically significant.

Overall, the results obtained using Fridericia’s correction are consistent with those obtained using Bazett’s correction, supporting the robustness of the observed increase in STV preceding arrhythmic events.

Figure S2. Comparison of STV values calculated using Fridericia’s QT correction across baseline recordings from Group 1 (G1 Baseline), baseline recordings from Group 2 (G2 Baseline), and arrhythmic event recordings from Group 2 (G2 Event). STV values were computed after exclusion of noisy and abnormal beats. Statistical significance is indicated as follows: n.s., p > 0.05; ** p ≤ 0.01; *** p ≤ 0.001.

| **Comparison** | **STV Condition A** | **STV Condition B** | **p-value** |
| --- | --- | --- | --- |
| BL2 vs Event | 3.2 (2.1–5.9) | 4.3 (2.2–6.5) | 0.0007 |
| BL1 vs BL2 | 3.2 (2.0–6.1) | 3.2 (2.1–5.9) | 0.9372 |
| BL1 vs Event | 3.2 (2.0–6.1) | 4.3 (2.2–6.5) | 0.0074 |

Table S2. Comparison of STV values calculated using Fridericia’s QT correction across study groups. Results are presented as median (IQR). Paired comparisons (Group 2 baseline vs event) were performed using the Wilcoxon signed-rank test, and unpaired comparisons using the Mann–Whitney U test.

# 3. ****Longitudinal Baseline STV Trends****

## ****Supplementary Methods****

To evaluate whether longitudinal changes in baseline STV could be associated with arrhythmic risk, we performed an exploratory analysis of STV trajectories across consecutive baseline transmissions.

For each transmission, STV values were computed over 140-second recordings using the same preprocessing pipeline described in Section 2.4, including removal of abnormal beats and exclusion of segments affected by excessive noise.

For each baseline transmission, an average STV value was calculated. To enable longitudinal analysis, only patients with at least three baseline transmissions were included. The resulting cohort consisted of 32 patients with subsequent arrhythmic events and 1391 patients without events.

STV trajectories were aligned relative to the time of the first baseline recording and expressed as days since baseline. Mean trajectories were computed for each group (patients with and without events), and variability was represented using interquartile ranges.

To compare longitudinal STV trajectories between groups, the area under the curve (AUC) was computed for each patient and compared using the Mann–Whitney U test

## ****Supplementary Results****

The longitudinal evolution of baseline STV values is shown in Figure S3. Mean STV trajectories were largely overlapping between patients who subsequently experienced arrhythmic events and those who did not. No clear separation in temporal patterns or consistent divergence between groups was observed over time. Although greater variability was observed in the group of patients with events, this is attributable to the smaller sample size. These fluctuations did not result in a sustained or progressive difference between the groups.

These findings suggest that longitudinal STV trends under baseline conditions do not provide additional discriminatory value for long-term risk stratification in this dataset, consistent with the absence of differences observed in single baseline STV measurements. Accordingly, comparison of the AUC between groups did not show a statistically significant difference (Mann–Whitney U test, p = 0.1410).

Figure S3. Longitudinal evolution of baseline STV values across consecutive transmissions in patients with and without subsequent arrhythmic events. Each curve represents the mean STV per transmission, computed over standardized segments after exclusion of noisy and abnormal beats. Shaded regions indicate interquartile ranges. No clear separation between groups is observed over time.
